# The association of statins and taxanes: an efficient combination trigger of cancer cell apoptosis

**DOI:** 10.1038/bjc.2012.6

**Published:** 2012-01-31

**Authors:** J Follet, L Corcos, G Baffet, F Ezan, F Morel, B Simon, C Le Jossic-Corcos

**Affiliations:** 1 Inserm U613-ECLA Team, Faculté de Médecine, Université Européenne de Bretagne, Université de Bretagne Occidentale, IFR 148-ScInBioS, 22 Avenue Camille Desmoulins, 29200 Brest, France; 2 CHU Brest, 5 Avenue Foch, 29200 Brest, France; 3 EA SERAIC-IRSET, Faculté de Pharmacie, IFR 140, 2 Avenue du Pr Léon Bernard, 35000 Rennes, France

**Keywords:** statins, taxanes, gastric cancer

## Abstract

**Background:**

Cancer cell killing might be achieved by the combined use of available drugs. Statins are major anti-hypercholesterolemia drugs, which also trigger apoptosis of many cancer cell types, while docetaxel is a potent microtubule-stabilising agent.

**Methods:**

Here, we looked at the combined effects of lovastatin and docetaxel in cancer cells.

**Results:**

Whole transcriptome microarrays in HGT-1 gastric cancer cells demonstrated that lovastatin strongly suppressed expression of genes involved in cell division, while docetaxel had very little transcriptional effects. Both drugs triggered apoptosis, and their combination was more than additive. A marked rise in the cell-cycle inhibitor p21, together with reduction of aurora kinases A and B, cyclins B1 and D1 proteins was induced by lovastatin alone or in combination with docetaxel. The drug treatments induced the proteolytic cleavage of procaspase-3, a drop of the anti-apoptotic Mcl-1 protein, Poly-ADP-Ribose Polymerase and Bax. Strikingly, docetaxel-resistant HGT-1 cell derivatives overexpressing the *MDR-1* gene were much more sensitive to lovastatin than docetaxel-sensitive cells.

**Conclusion:**

These results suggest that the association of lovastatin and docetaxel, or lovastatin alone, shows promise as plausible anticancer strategies, either as a direct therapeutic approach or following acquired P-glycoprotein-dependent resistance.

Gastric cancer is mostly associated with poor survival and ranks five in Europe in terms of incidence (Ferlay *et al*, 2010); it is also one major cause of cancer-related death worldwide. Because gastric cancer is often detected at late stages, available treatments are mostly inefficient (Shah and Kelsen, 2010). Microtubule targeting agents of the taxane class, like docetaxel, have been used for almost a decade to treat refractory breast cancer, and they have also been foreseen to treat gastric cancer. However, only few patients could benefit from docetaxel treatment, mostly because of severe side effects (Baker *et al*, 2009; Nishiyama and Wada, 2009). Nevertheless, many clinical trials recorded at the NIH plan to evaluate docetaxel as an anticancer agent in combination with other chemotherapeutic agents, including 5-fluorouracile, cisplatin, epirubicin, or bevacizumab, among several others (Ajani, 2008; Di Lauro *et al*, 2009; Goel *et al*, 2010; Smith *et al*, 2011). Hence, docetaxel is believed to be promising for the treatment of gastric cancer.

Statins are widely used as anti-hypercholesterolemia drugs, but they also bear potential as either cancer preventive or adjuvant therapies (Demierre *et al*, 2005; Sassano and Platanias, 2008). There has been some dispute in the literature concerning the chemoprevention statins may exert on cancer occurrence (Bjerre and LeLorier, 2001; Katz, 2005). By contrast, analyses in experimental models have mostly converged to suggest that statins may increase the efficacy of cancer cell killing by several classes of drugs and used to target various types of cancer cells (Graaf *et al*, 2004; van der Spek *et al*, 2009; Sane *et al*, 2010; Zheng *et al*, 2010). Hence, combinations of statins with DNA damaging agents like topoisomerase inhibitors, cisplatin, or 5-fluorouracile have shown increased cell death, reduced tumour growth, or metastases (Feleszko *et al*, 1998; Agarwal *et al*, 1999; Wang *et al*, 2002; Kozar *et al*, 2004).

The molecular mechanisms evoked in response to statins or docetaxel have been investigated in cell culture and animal models. Docetaxel promotes microtubulin assembly and stabilises the polymers against depolymerisation, thereby inhibiting microtubule dynamics (Ringel and Horwitz, 1991). As a result, mitotic progression is restricted. Statins have no reported effects on the activity of the mitotic spindle. Docetaxel triggers the degradation of the anti-apoptotic Bcl-2 protein through increased phosphorylation (Berchem *et al*, 1999). Similarly, statins suppress the Bcl-2 protein (Wong *et al*, 2002). Both docetaxel and statins inhibit angiogenesis, although this may depend on the administered dose for statins (Sweeney *et al*, 2001; Skaletz-Rorowski and Walsh, 2003). Statins can induce the cell-cycle inhibitor protein p21, which is also true for docetaxel (Gray-Bablin *et al*, 1997; Lee *et al*, 1998; Gan *et al*, 2011; Shin *et al*, 2011). The combination of paclitaxel and lovastatin had a synergistic effect on apoptosis of leukaemia cells (Holstein and Hohl, 2001), whereas the same combination did not show any increase in cytotoxicity in anaplastic thyroid carcinoma cells (Chung *et al*, 2011). The combination of docetaxel, which is more potent than paclitaxel, with statins has not been tested.

This study was devised to address the question of the effects of such a combined treatment in the human gastric HGT-1 cancer cell line. The results presented herein show that the association of lovastatin and docetaxel provided a more than additive apoptotic response. In addition, HGT-1-derived docetaxel-resistant cells were sensitive to statins, even more than parental HGT-1 cells. As a whole, this novel drug combination was more efficient at inducing apoptosis than either drug alone in HGT-1-sensitive cells. Lovastatin was, by itself, able to overcome the acquired resistance to docetaxel.

## Materials and methods

### Cell culture

HGT-1 human gastric cancer cells were grown at 37°C under a humidified atmosphere of 5% CO_2_ in DMEM (Dulbecco's modified Eagle's medium) (Lonza, Saint Beauzire, France), containing 4.5 g L^−1^ glucose and supplemented with 5% fetal bovine serum without antibiotics (Gibco-Invitrogen, Cergy-Pontoise, France) (Laboisse *et al*, 1982; Gibot *et al*, 2009). HepG2 human hepatoblastoma (DMEM/F12, 1/1, 5% fetal bovine serum), HeLa human cervical cancer (DMEM, 10% fetal calf serum), and H322 human lung cancer (RPMI-1640, 10% fetal calf serum) cells were also used (Supplementary Figure 2). All cell lines were free of mycoplasma.

Selection of the docetaxel-resistant cell populations from HGT-1 cells was performed with mass cultures grown in complete medium supplemented with 5 nM docetaxel. Massive cell death occurred for several weeks under continuous selective pressure, after which the populations stabilised and started to grow with no more signs of death. Cells were constantly grown in the presence of 5 nM docetaxel, except for the cell passage that preceded the experiments that involved drug treatments.

### Fluorescence *in-situ* hybridisation

Fluorescence *in-situ* hybridisation (FISH) was used to determine the number of *MDR1* chromosomal copies using bacterial artificial chromosome (BAC) clones chosen from the human genome browser database of the Genome Bioinformatics Group at the University of California, Santa Cruz (http://www.genome.ucsc.edu/). BACs RP11-806M4 and RP11-42N21 were extracted using standard methods and then labelled by nick translation in spectrum orange (Abbott, Rungis, France) and in spectrum green (Abbott), respectively. Dual FISH using RP11-42N21 and RP11-806M4 was performed on HGT-1 and HGT-1-D5 cell lines according to the standard procedures (Morel *et al*, 2003). After hybridisation, the slides were counterstained with 4′,6-diamidino-2-phenyl-indole and analysed with a Zeiss Axio Plan microscope (Carl Zeiss SAS, Le Pecq, France). Subsequent image acquisition was performed using a CCD camera with Isis (MetaSystems, Altlussheim, Germany). For each cell line, at least 30 metaphases were analysed.

### Determination of apoptotic chromatin fragmentation

The cells were treated with different concentrations of docetaxel, lovastatin or with both. Apoptosis was determined by Hoechst 33342 (10 *μ*g ml^−1^ in phosphate buffered saline (PBS)) staining of the cells for 15 min at 37°C and fluorescence microscopy analysis of 300 cells per condition, from triplicate cultures.

### RNA extraction and reverse transcription–PCR analysis

Total RNA was isolated using Trizol (Invitrogen, Cergy-Pontoise, France). The integrity and purity of the RNA was determined by measuring the optical density ratio (A260/A280) and the RIN (RNA Integrity Number) using the RNA 6000 Nano LabChip (Agilent, Massy, France) and the Agilent 2100 bioanalyzer (Agilent). The RNA samples were used for the first-strand cDNA synthesis with the High Capacity cDNA Reverse Transcription kit and random hexamer primers (Applied Biosystems, Courtaboeuf, France). Quantitative real-time reverse transcription (RT)–PCR was performed using the Power SYBR Green Kit (Applied Biosystems) according to manufacturer's instructions with an ABI 7300 real-time PCR system (Applied Biosystems). mRNA levels were analysed in duplicate, normalised against GAPDH or phospho-protein P0 as an internal control gene. The results are expressed as the relative gene expression using the ΔΔCt method (Livak and Schmittgen, 2001). All of the tested genes were selected based on the microarray analyses (see below). In particular, we have validated and further studied: HMGCR (encoding HMG-CoA reductase), farnesyl pyrophosphate synthase (FPPS) (also known as FDPS, Supplementary Table 2), Sterol Element Binding Protein (SREBP)-1 and SREBP-2, low-density lipoprotein receptor (LDL-R), cyclins B1 and D1, aurora kinases A and B, survivin, p21 and p27 (Supplementary Table 3). The primer sequences and reaction conditions will be provided upon request.

### Microarray analysis

Double-stranded cDNA was synthesised from 500 ng of total RNA using the Quick Amp Labeling Kit, One-color, as instructed by the manufacturer (Agilent). Labelling with cyanine3-CTP, fragmentation of cRNA, hybridisation using the whole human genome kit (4 × 44K), and washing were performed according to manufacturer's instructions (Agilent). The microarrays were scanned and the data were extracted with the Agilent Feature Extraction Software. GeneSpring GX 11.0 (Agilent) was used to compare the data from treatment conditions with control. Three independent experiments were used for this microarray analysis.

### Protein extraction and western blotting analysis

The cells were harvested, washed in PBS, and lysed in RIPA buffer (50 mM Tris–HCl pH 7.4, 150 mM NaCl, 0.5% sodium deoxycholate, 0.1% SDS, 1% NP-40, 1 mM EDTA, and 1 mM PMSF) containing protease inhibitor cocktail (Roche, Meylan, France) and phosphatase inhibitor (Active Motif, Rixensart, Belgium) for 10 min at 4°C. Sixty micrograms of proteins were boiled in Laemmli sample buffer (Bio-Rad, Marnes la Coquette, France) for 5 min, separated by SDS–PAGE using 12% or 15% polyacrylamide gels and blotted onto polyvinyl difluoride membranes (GE Healthcare, Orsay, France). Non-specific binding sites were blocked for 1 h at room temperature by 5% (wt/v) fat-free milk before overnight incubation at 4°C with specific rabbit (or mouse for cyclin B1) anti-human antibodies: aurora kinases A and B, procaspase-3, PARP, Bcl-2, Bax, and survivin (Cell Signaling Technology, Ozyme, Saint Quentin en Yvelines, France), p27, p21, Mcl-1, cyclin B1 (Santa Cruz Biotechnology, Tebu-bio, Le Perray-en-Yvelines, France), cyclin D1 (NeoMarkers, Thermo Fisher Scientific, Illkirch, France), or HSC70 (Abcam, Paris, France) as a loading control. Anti-phospho-ERK1/2 was a mouse monoclonal antibody against a synthetic phosphopeptide (residues around threonine 202 and tyrosine 209 of human p44 MAPK; Cell Signaling Technology, Ozyme). Polyclonal antibodies against ERK1 (rabbit, sc-94) or ERK2 (rabbit, sc-154) and phospho-MEK1/2 were from Santa Cruz Biotechnology (Tebu-bio). Rabbit anti-phospho-JNK was from Cell Signaling and rabbit polyclonal anti-P38 antibody from Santa Cruz Biotechnology (Tebu-bio). Antibodies were used at the following dilutions: 1 : 500 (cyclin D1, Mcl-1, cyclin B1, p27, p21, Bax, ERK1/2, phospho-MEK1/2, p38, phospho-JNK, phospho-ERK1/2), 1 : 1000 (Caspase-3, PARP, aurora kinases A and B, survivin), and 1 : 2000 (Hsc70). Primary antibodies were detected with horseradish peroxidase-conjugated IgGs (1 : 5000; GE Healthcare). Blots were revealed using an Enhanced Chemiluminescence detection kit (GE Healthcare) by the Chemi-Capt 5000 software (Vilber Lourmat, Marne la Vallée, France).

## Results

### 1-Lovastatin, docetaxel, and combinations of both trigger HGT-1 apoptosis

We have shown previously that lovastatin could induce apoptosis of HGT-1 gastric cancer cells in a dose- and time-dependent manner (Gibot *et al*, 2009). Pilot experiments were performed to determine what would be the best docetaxel concentrations to evaluate its effects (Supplementary Figure 1). We selected the concentrations of 12.5 *μ*M lovastatin and 5 or 10 nM docetaxel for 48 h.

**Figure 1:**
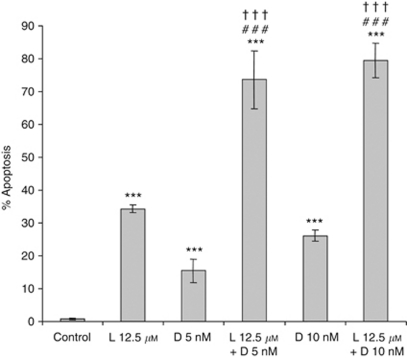
Apoptosis induction by lovastatin and docetaxel in HGT-1 gastric cancer cells. HGT-1 cells were treated with 12.5 *μ*M lovastatin (L12.5) or with 5 or 10 nM docetaxel (D5 or D10) alone or in combination for 48 h. Apoptosis was determined by Hoechst 33342 staining. Values are means ±s.d. (*n*=6). ^***^Compared with control, ^###^compared with docetaxel treatment, ^†††^compared with lovastatin treatment (*P*<0.001, Student's *t*-test).

As shown in Figure 1, 35% apoptosis was attained in response to 12.5 *μ*M lovastatin for 48 h. Docetaxel also induced apoptosis, although at a lower level (15% and 27% for 5 and 10 nM, respectively). That docetaxel-induced apoptosis was further demonstrated by the ability of the broad spectrum caspase inhibitor Z-VAD-*fmk* to suppress cell death (data not shown). Strikingly, the exposure to both drugs had a more than additive effect on apoptosis (up to 80% apoptosis), when compared with the effect expected from the addition of apoptosis % obtained for the drugs used alone (50% and 60.5% for lovastatin+docetaxel 5 nM and lovastatin+docetaxel 10 nM, respectively, *P*<0.001; Student's *t*-test). Hence, docetaxel induced apoptosis in these gastric cancer cells, and this effect was strongly enhanced by lovastatin. This drug combination (12.5 *μ*M lovastatin+5 nM docetaxel) also triggered apoptosis in other cell types, including HepG2 human hepatoblastoma, HeLa cervical cancer, and H322 lung cancer cells, as demonstrated by increased caspase 3/7 activity (Supplementary Figure 2).

### 2-Microarray analyses reveal a wealth of changes induced by lovastatin in HGT-1 cells

To test the effects of the drugs on gene expression profiles, HGT-1 cells were treated by 12.5 *μ*M lovastatin, 5 nM docetaxel, or by a combination of both for 48 h. RNA was extracted and used for whole transcriptome analysis with 44k Agilent gene chips in triplicate experiments. Lovastatin induced 362 genes (two-fold variation, *P*<0.01) and repressed 508 genes (Supplementary Table 1). In sharp contrast, at these levels of confidence and threshold, docetaxel did not modify expression of any gene. The drug combination led to higher numbers of upregulated (499) and downregulated (552) genes than attained with lovastatin alone, indicating that both drugs interacted under these conditions. This suggests that the stronger apoptosis-inducing effect of the drug combination might result, in part, from this additional set of gene expression modifications.

Lovastatin induced many genes involved in lipid biosynthesis pathways (1.6- to 4.4-fold increases; Supplementary Table 2). By contrast, lovastatin strongly suppressed expression of many genes involved in cell division and cell-cycle progression, while increasing the *p21* gene that encodes a cell-cycle repressor protein (Supplementary Table 3).

### 3-Lipid synthesis control is impaired in lovastatin-treated cells

**Figure 2:**
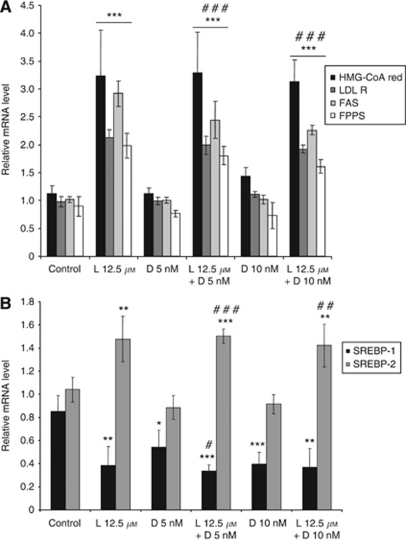
Effect of lovastatin and docetaxel on lipid synthesis gene expression levels. HGT-1 cells were treated with 12.5 *μ*M lovastatin (L12.5) or with 5 or 10 nM docetaxel (D5 or D10) alone or in combination for 48 h. HMG-CoA reductase (HMG-CoA red), LDL receptor (LDL-R), fatty acid synthase (FAS), FPP synthase (FPPS) (**A**) and SREBP-1 and SREBP-2 (**B**) mRNA levels were analysed by real-time RT–PCR (see Materials and Methods). Relative mRNA levels were normalised to P0 mRNA levels. Values are means±s.d. (*n*=4). ^*^Compared with control and ^#^compared with docetaxel treatment. One symbol: *P*<0.05, two symbols: *P*<0.01, three symbols: *P*<0.001 (Student's *t*-test).

To characterise in more details the effects of the drugs on lipid synthesis genes, HGT-1 cells were treated by either lovastatin or docetaxel, or by combinations of both for 48 h. Relative mRNA levels were determined by quantitative real-time RT–PCR. As shown in Figure 2A, the LDL-R, the HMG-CoA reductase, the FPPS, and the fatty acyl synthase (FAS) genes were all induced by lovastatin, but not by docetaxel, confirming the microarray results. The exposure to both drugs showed inductive effects similar to those obtained for lovastatin alone.

By contrast, although expression of the *SREBP-2* gene was induced by lovastatin (which was not modified in the presence of docetaxel that had no effect by itself), expression of SREBP-1 was significantly reduced by all treatments (Figure 2B). Such a duality of effects on either SREBP transcript indicates that, while SREBP-2 was increased as part of the positive regulatory feedback evoked by shortage of the mevalonate pathway, the *SREBP-1* gene behaved like a negative stress response gene. Expression of the *SREBP-1* gene was not detected in the microarray analysis.

### 4-Proteolytic cleavage of apoptosis proteins in response to drug treatments

**Figure 3:**
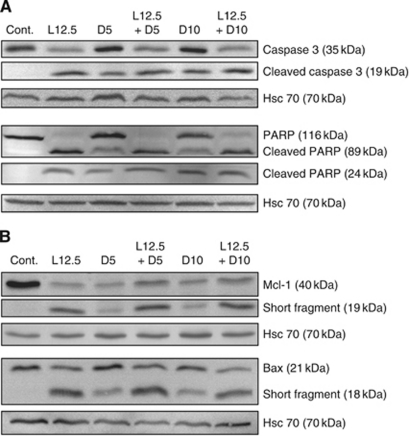
Effect of lovastatin and docetaxel on caspase-3, Poly-ADP-Ribose Polymerase (**A**), Mcl-1 and Bax protein levels (**B**). HGT-1 cells were treated with 12.5 *μ*M lovastatin (L12.5) or with 5 or 10 nM docetaxel (D5 or D10) alone or in combination for 48 h. Protein levels were analysed by western blotting. Hsc70 was used as a loading control. The results are representative of three experiments with similar results.

Procaspase-3 and Poly-ADP-Ribose Polymerase were cleaved in response to lovastatin and docetaxel or combination of both drugs, further demonstrating apoptosis engagement (Figure 3A). Procaspase-7 was also cleaved, especially for the highest drug concentrations (data not shown).

All treatments triggered suppression of the major anti-apoptotic Mcl-1 protein (Figure 3B). A short 19-kDa fragment appeared, resulting from apoptosis, as z-VAD-*fmk* prevented its appearance (data not shown). Bax, a major pro-apoptotic member of the Bcl-2 protein family, was either slightly suppressed in presence of lovastatin or combination of the two drugs, or remained unchanged in response to docetaxel. A short 18-kDa fragment also appeared in response to the treatments, mainly in the presence of lovastatin, alone or combined, and may have been induced by caspases activity as it disappeared under treatments combined to z-VAD-*fmk* (data not shown).

### 5-Cell cycle and mitosis impairment

**Figure 4:**
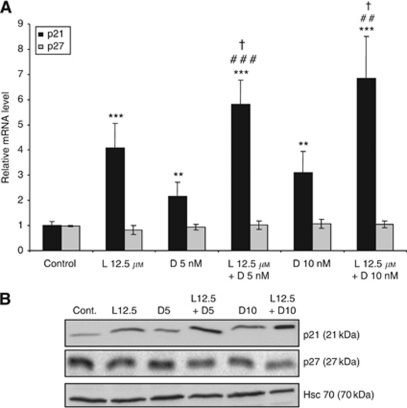
Effect of lovastatin and docetaxel on p21 and p27 expression. HGT-1 cells were treated with 12.5 *μ*M lovastatin (L12.5) or with 5 or 10 nM docetaxel (D5 or D10) alone or in combination for 48 h. (**A**) p21 and p27 mRNA levels were analysed by real-time RT–PCR. Relative mRNA levels were normalised to GAPDH mRNA levels. Values are means±s.d. (*n*=4). ^*^Compared with control, ^#^compared with docetaxel treatment, †compared with lovastatin treatment. One symbol: *P*<0.05, two symbols: *P*<0.01, three symbols: *P*<0.001 (Student's *t*-test). (**B**) p21 and p27 protein levels were analysed by western blotting. Hsc70 was used as a loading control. The western blotting analyses are representative of three experiments with similar results.

Since docetaxel hampers mitosis, we sought to determine the effects of lovastatin and docetaxel on p21 and p27 transcript levels. As observed in microarray experiments, the p21 transcript was induced by lovastatin (Supplementary Table 3). As shown in Figure 4A, both drugs induced p21 expression, with a more marked effect (up to four-fold) with lovastatin than for docetaxel. The drug combination led to an effect higher (up to seven-fold for 10 nM docetaxel+lovastatin) than obtained for the drugs used as single agents. These increases in the p21 transcript were associated with a parallel increase in the p21 protein (Figure 4B). The expression of p27 was slightly reduced by the drug combination (Figure 4B).

**Figure 5:**
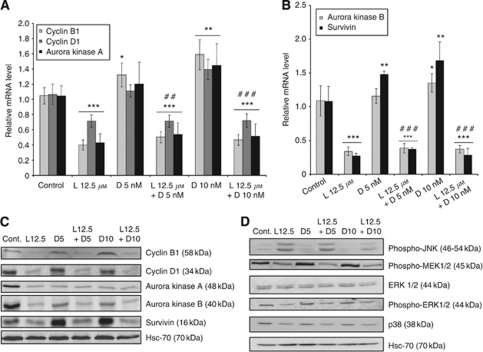
Effect of lovastatin and docetaxel on expression of genes involved in the initiation and progression of mitosis, cytokinesis, and MAP kinases signalling pathway. HGT-1 cells were treated with 12.5 *μ*M lovastatin (L12.5) or with 5 or 10 nM docetaxel (D5 or D10) alone or in combination for 48 h. Cyclin D1, cyclin B1, aurora kinase A (**A**), aurora kinase B and survivin (**B**) mRNA levels were analysed by real-time RT–PCR. Relative mRNA levels were normalised to P0 mRNA levels. Values are means±s.d. (*n*=4). ^*^Compared with control, #compared with docetaxel treatment, ^†^compared with lovastatin treatment. One symbol: *P*<0.05, two symbols: *P*<0.01, three symbols: *P*<0.001 (Student's *t*-test). (**C**) Cyclin B1, cyclin D1, aurora kinase A, aurora kinase B, and survivin protein levels were analysed by western blotting. Hsc70 was used as a loading control. (**D**) phospho-JNK, phospho-MEK1/2, ERK1/2, phospho-ERK1/2, and p38 protein levels were analysed by western blotting. Hsc70 was used as a loading control. The western blotting analyses are representative of three experiments with similar results.

To analyse the effects of the drugs on proteins involved in mitosis progression, we next looked at expression of cyclin B1, cyclin D1, and aurora kinase A. As shown in Figure 5A, lovastatin reduced expression of all transcripts, both alone, in agreement with microarray experiments, or when combined with docetaxel. In addition, lovastatin repressed aurora kinase B and survivin, even more strongly than cyclin B1, cyclin D1, and aurora kinase A mRNAs, either alone or combined with docetaxel, although docetaxel induced these transcripts (mostly survivin) (Figure 5B). In addition, lovastatin alone or in combination with docetaxel triggered a decrease in all proteins. Docetaxel also triggered a decrease in aurora kinases A and B, but slightly increased cyclin B1 (Figure 5C). Strikingly, docetaxel strongly induced survivin levels. However, lovastatin blocked this inducing effect, and even suppressed the protein in the combination of both drugs at the highest concentration.

Finally, we analysed expression of the jun kinase (JNK), p38 and ERK pathways in response to lovastatin and docetaxel (Figure 5D). The phosphorylated form of JNK was induced in the presence of lovastatin. Conversely, the levels of phosho-MEK1/2 and phosho-ERK1/2 were decreased by lovastatin, similarly to p38 MAP kinase. We also demonstrate that docetaxel had no effect on these signalling pathways, and did not modify the effects of lovastatin when used in combination.

### 6-Effect of the vinblastine/lovastatin combination on cell death

As a control for the specificity of docetaxel, we used vinblastine, which acts in the reverse way as docetaxel by inhibiting the re-polymerisation of microtubules. Vinblastine (1 nM, 48 h) triggered about 30% apoptosis but did not add to the effect of lovastatin, even showing some antagonism over the expected death rate (for the 1 nM drug concentration; Supplementary Figure 3). Hence, these observations clearly distinguished docetaxel from vinblastine, despite the fact that both drugs could efficiently trigger apoptosis of HGT-1 cells.

### 7-Isolation of docetaxel-resistant cells

Drug resistance is a serious hurdle for the treatment of cancer patients. In order the look for novel ways to get around acquired resistance to docetaxel, we isolated a population derived from HGT-1 cells (named HGT-1-D5) following selection in the continuous presence of 5 nM docetaxel. The HGT-1-D5 cell population was also resistant to vinblastine-induced apoptosis. This resistance was fully overcome in the presence of verapamil (Supplementary Figure 4), a P-glycoprotein (Pgp, the product of the *MDR-1* gene) blocker, indicating that one major difference between HGT-1 and HGT-1-D5 cells was at the level of expression and function of Pgp. To verify that HGT-1-D5 cells overexpressed the *MDR-1* gene, we performed quantitative RT–PCR with MDR-1 primers. This analysis showed that the *MDR-1* gene was dramatically overexpressed in HGT-1-D5 cells, whereas no signal could be obtained with the parental cells (data not shown). In addition, the expression of the other members of the ABC transporters (MRP-1/2/3) was unchanged (data not shown). Hence, these results demonstrate that the acquired resistance to docetaxel of HGT-1-D5 cells was due to a massive overexpression of Pgp. To fully confirm these data, we performed a FISH analysis using probes that cover the *MDR-1* gene locus. Chromosome amplification was readily detected in 47.5% of mitotic figures of HGT-1-D5 but not HGT-1 cells, in support of the overexpression of MDR-1 transcript in HGT-1-D5 cells (Supplementary Figure 5).

### 8-Enhanced apoptosis by lovastatin in HGT-1-D5 cells

**Figure 6:**
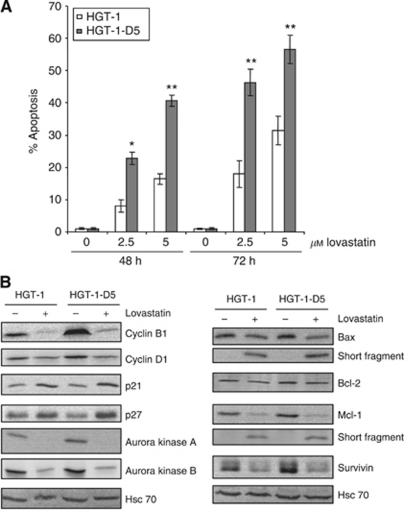
Comparison of the effect of lovastatin in HGT-1 and HGT-1-D5 cells. HGT-1 and HGT-1-D5 cells were treated with 0, 2.5, or 5 *μ*M lovastatin for 48 or 72 h (**A**). Apoptosis was determined by Hoechst 33342 staining. Values are means±s.d (*n*=3). ^*^P<0.1 or ^**^P<0.01 for HGT-1-D5 cells compared with HGT-1 cells (Student's *t*-test). (**B**) HGT-1 and HGT-1-D5 cells were treated with 2.5 *μ*M lovastatin for 72 h. Protein levels were analysed by western blotting. Hsc70 was used as a loading control. The western blotting analyses are representative of three experiments with similar results.

To determine the effect of lovastatin on HGT-1-D5 cells, we treated the cells with either 2.5 or 5 *μ*M lovastatin, that is, drug concentrations lower than those used for the initial part of the study, for 48 or 72 h. As shown in Figure 6A, both concentrations triggered a dose- and time-dependent increase in apoptosis in HGT-1 cells (up to 32%). Strikingly, HGT-1-D5 cells were much more sensitive to lovastatin than HGT-1 cells (up to 55% apoptosis). To further characterise the docetaxel-resistant cells, we performed western blotting analyses. As shown in Figure 6B, protein levels were comparable between HGT-1 and HGT-1-D5 cells, except for cyclin B1 and survivin, which were increased in HGT-1-D5 cells. Lovastatin reduced strongly (aurora kinases, Mcl-1) or more weakly (cyclin D1, Bax), or induced (p21) protein levels similarly in HGT-1 and HGT-1-D5 cells. Overexpression of survivin and cyclin B1 proteins in HGT-1-D5 cells was fully suppressed by lovastatin. In addition, lovastatin slightly induced p27 in HGT-1-D5 cells, adding a further level of cell-cycle blockade. Even though cell cycle-associated cyclin B1 or survivin was expressed at higher levels in HGT-1-D5 cells than in HGT-1 cells, this was abolished by lovastatin treatment.

## Discussion

The cytotoxic activity of docetaxel has been attributed to its ability to stabilise the mitotic spindle, upon blocking microtubule depolymerisation. Direct consequences of this activity were a block of the cell cycle in the G2/M transition (Fabbri *et al*, 2006), or in subG1 (Hernandez-Vargas *et al*, 2007). Quite often, this was associated with an increase in p21 (Fabbri *et al*, 2008). In addition, overexpression of p21 in docetaxel-resistant cells restored drug sensitivity (Canfield *et al*, 2006). Interestingly, survivin gene expression was inducible by docetaxel in DU145 human prostate cancer cells (Kim *et al*, 2006), much like we observed in HGT-1 cells. This observation could seem surprising, in view of the death potential of docetaxel treatment. However, it was shown that the increase in survivin was associated with the nuclear interaction with the pro-apoptotic Smac/DIABLO protein, which was proposed to increase cell death in this model (Kim *et al*, 2006). In addition, cyclin B1 was able to promote docetaxel-induced apoptosis (Gomez *et al*, 2007), an effect that could participate in the induction of HGT-1 apoptosis, as cyclin B1 expression was slightly induced by docetaxel. Aurora kinases A and B proteins were reduced by docetaxel treatment of HGT-1 cells, a finding in good agreement with previous studies that have clearly shown that inhibition of either kinase enhanced the cell killing activity of taxanes (Tanaka *et al*, 2007).

Drug combinations with taxanes have been reported to increase cell killing, as compared with the effects of single agents, either by combining dual cell death promoting activities (Bijnsdorp *et al*, 2008; Reiner *et al*, 2009) or by adding a drug efflux blocking activity to a cytotoxic effect (Miettinen *et al*, 2009). The interplay between microtubule poisoning and impairment of the mevalonate pathway, as analysed in this study, also proved to be remarkably efficient to induce high levels of apoptotic cell death. These events were independent on p53 as HGT-1 cells carry a mutation in the gene (Sadji-Ouatas *et al*, 2002). Our studies could be extended to gastric cancer cell lines that are p53 proficient, as roughly 65% of p53 proteins in gastric cancers should be wild type (Lane, 2005). Importantly, experiments conducted in HepG2 (hepatoblastoma) and HeLa (cervical cancer) cells – both carrying wild-type p53 – showed strong increases in caspase-3/7 activation following combined treatment with lovastatin and docetaxel (Supplementary Figure 2). In addition, such increases in caspase-3/7 activation were also observed in H322 (p53 deleted, lung cancer) cells (Supplementary Figure 2).

Our results showed that, while lovastatin-induced massive changes in gene expression, docetaxel had rather limited effects. The addition of both drugs, by enlarge, gave the same repertoire of gene changes as lovastatin alone, although some additional gene expression changes were obtained by the combined treatments. As expected, lovastatin induced the SREBP-dependent gene battery. This was anticipated and was likely the result of activation of the positive feedback control mechanism that involves sterol regulatory element binding proteins (SREBP), which *trans*-activate these genes when mevalonate shortage occurs (Sakakura *et al*, 2001). In addition, lovastatin also triggered downregulation of genes involved in the control of cell division. Under our conditions, however, docetaxel had no or only little effect on gene expression at the threshold value of two-fold variation, further indicating that docetaxel-induced apoptosis mostly resulted from its direct activity onto the mitotic spindle.

At the cell level, docetaxel induced apoptosis, an effect synergistic with that of lovastatin, a potent apoptosis inducer in these cells. The links between gene expression remodelling and engagement of the apoptotic program may be plenty. However, as lovastatin suppressed the pro-survival mevalonate pathway, it is likely that mevalonate shortage had a major role in induced cell death, in spite of the stimulation of the genes from the *SREBP* gene battery as a rebound mechanism, an inductive response mostly unproductive since mevalonate synthesis was continuously blocked in the presence of lovastatin. Among the cell targets that may be most affected by lovastatin are the Ras, Rho, or Rab proteins that need prenylation (the post-translational addition of C15 or C20 lipid moieties to the C-terminal end of a limited subset of proteins) to anchor to the plasma membrane and gain biochemical activities (Demierre *et al*, 2005). This will have to be investigated in future experiments.

Both docetaxel and lovastatin suppressed the anti-apoptotic Mcl-1 protein, induced the cell-cycle inhibitor p21 mRNA and protein, and even stronger effects were obtained upon addition of both compounds. The cleavage product of the Mcl-1 protein might correspond to the one described by Cartron *et al* (2004), which could behave like a pro-apoptotic BH3-only protein. Docetaxel slightly induced cyclin B1 mRNA and protein, weakly induced cyclin D1 mRNA – but not protein, slightly increased aurora kinases mRNA, but suppressed the proteins. This could be explained by the fact that, because of cell cycle blockade, the aurora kinases could be degraded at that late (48 h) time point. Lovastatin suppressed cyclin B1, D1, aurora kinase A, aurora kinase B, and survivin mRNA levels. In addition, although docetaxel induced survivin mRNA and protein, this was abolished by lovastatin. Further experiments will be needed to explore more precisely the mechanisms involved in the repression of aurora kinases by lovastatin, an effect that has not been reported before. In addition, docetaxel induced survivin expression, despite triggering apoptosis. Therefore, it appears that docetaxel may have somewhat contradictory effects with respect to cell death and cell division control, as it may stimulate both pro-death and pro-survival pathways. By contrast, lovastatin opposed docetaxel to suppress survivin induction and promote cell death. Similarly, the caspase-mediated cleavage of Mcl-1 and Bax, mainly resulting from lovastatin treatment, alone or combined, could amplify the apoptotic response.

Finally, the phosphorylated form of JNK was induced by lovastatin, whereas the levels of phosho-MEK1/2 and phosho-ERK1/2 were decreased, similarly to p38 MAP kinase. We also demonstrate that docetaxel had no effect on JNK, p38 and ERK signalling pathways, and did not modify the effects of lovastatin when used in combination. These results were in good agreement with the ability of lovastatin to slow down cell-cycle progression and trigger cell death through induction of cell stress pathways, especially through JNK, but not p38, activation (Cerezo-Guisado *et al*, 2007; Liu *et al*, 2009).

As an approach to identify new ways to get around established resistance to docetaxel, we isolated an HGT-1 derivative cell line that was stably resistant to 5 nM docetaxel. This phenotype was due to the amplification of the *MDR-1* gene locus that encodes Pgp, a specific membrane transporter protein responsible for the expulsion of many drugs, restricting their active concentration within cells and thus cytotoxic activity. Strikingly, cyclin B1 and survivin proteins were more expressed in HGT-1-D5 cells than in HGT-1 cells. When treated with lovastatin at low concentrations (2.5 and 5 *μ*M), the resistant cells were shown to be exquisitely sensitive to apoptosis induction, significantly more than HGT-1 parental cells. Furthermore, p21 and p27 protein levels were induced by lovastatin in HGT-1-D5 cells. The responses of the other genes to the drugs were not different between HGT-1-D5 and HGT-1 cells. Hence, acquired resistance to docetaxel was not associated with a reduced sensitivity to lovastatin-induced apoptosis or to an inability of lovastatin to influence target protein expression. As a whole, these results demonstrate that it was possible to overcome efficiently an acquired resistance to docetaxel in human gastric cancer cells upon using lovastatin at concentrations that were close to those attainable in serum (Thibault *et al*, 1996). Importantly, the concentrations of docetaxel used in this model *in-vitro* system were far below what is used for cancer treatment, as clinically relevant concentrations of plasma docetaxel range between 1.5 and 6 *μ*M (Urien *et al*, 1996). By extension, our results might also apply to other chemotherapeutics-dependent resistance resulting from *MDR-1* gene overexpression.

This study brings in new lights into the mechanisms evoked by both docetaxel and lovastatin to reduce cell division and increase apoptotic cell death. Hence, it will be fair to assume that such a combination of compounds could offer new therapeutic options for the treatment of gastric cancer. Furthermore, our data suggest that, should resistance to anticancer agents due to a stable MDR-1 overexpression occur, this may be efficiently overcome through the use of lovastatin in adjuvant therapies. Moreover, the treatment of lovastatin-resistant HGT-1-derived cells (Gibot *et al*, 2009) by docetaxel triggered apoptosis at a higher level than for HGT-1 parental cells (data not shown). The same reasoning as above can be made: in case of ‘resistance’ to statins – as may have occurred over years of statin therapy, or inter-individual variability – the use of docetaxel could open new treatment options for human patients.

In summary, our study has shown the potential of the docetaxel+lovastatin combination for the efficient induction of human gastric cancer cell death, among other tumour types, and the ability of lovastatin to trigger apoptosis of cancer cells overexpressing the *MDR-1* gene. This may open the path to clinical trials for patients suffering from gastric cancers. The fact that statins are widely used in the human population without provoking deleterious effects would make this strategy readily acceptable.
