# Supplementary material for: The association of statins and taxanes: an efficient combination trigger of cancer cell apoptosis
Source: Br J Cancer. 2012 Jan 31;106(4):685–92. doi: 10.1038/bjc.2012.6 (PMC3322964; doi:10.1038/bjc.2012.6)
Supplement: Supplementary Figures [file bjc20126x1.ppt]

## Slide 1
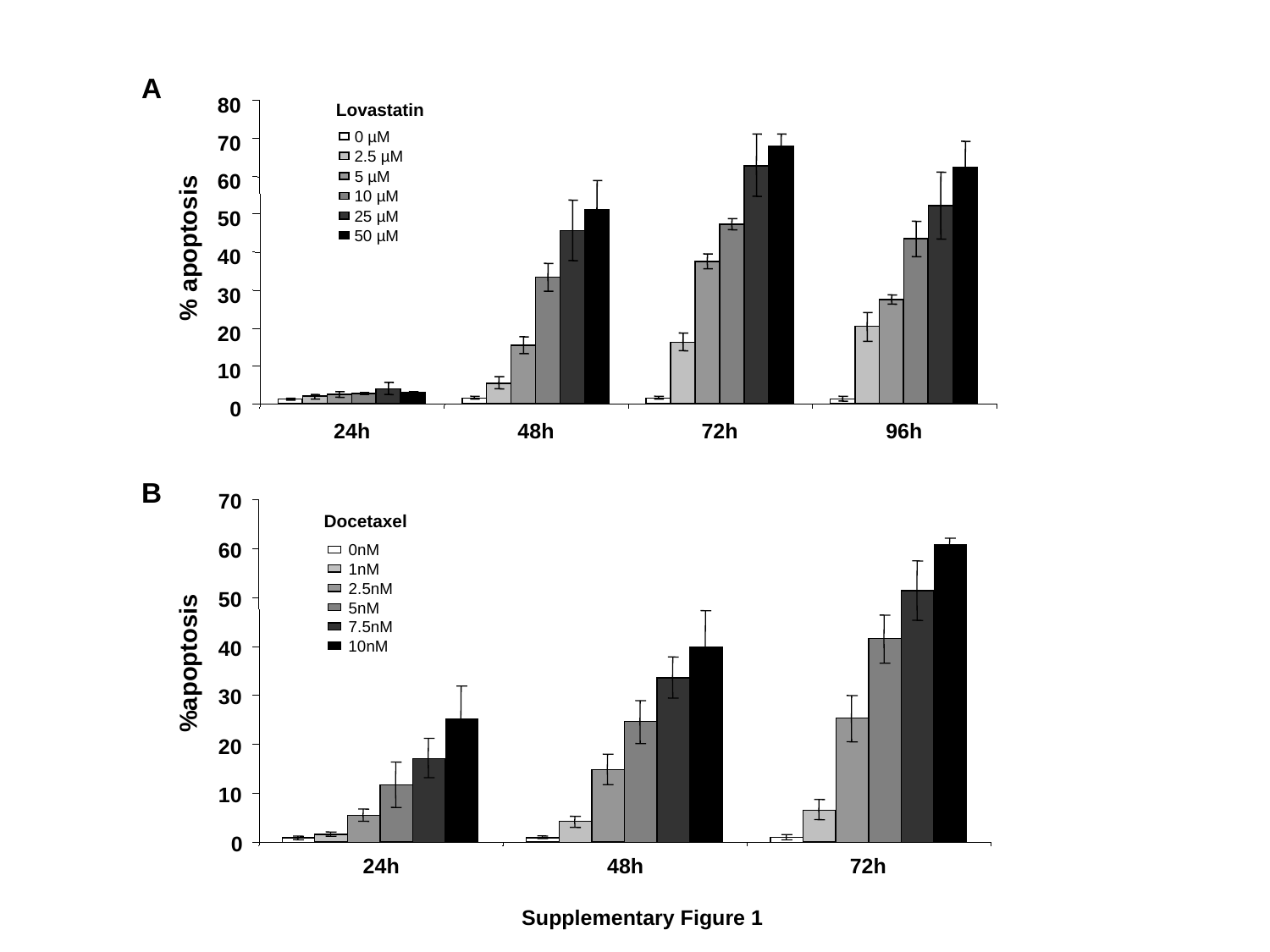

A
80
70
60
50
40
30
20
10
0
Lovastatin
0 µM
2.5 µM
5 µM
10 µM
25 µM
50 µM
% apoptosis
24h
48h
72h
96h
B
70
60
50
40
30
20
10
0
Docetaxel
0nM
1nM
2.5nM
5nM
7.5nM
10nM
%apoptosis
24h
48h
72h
Supplementary Figure 1

## Slide 2
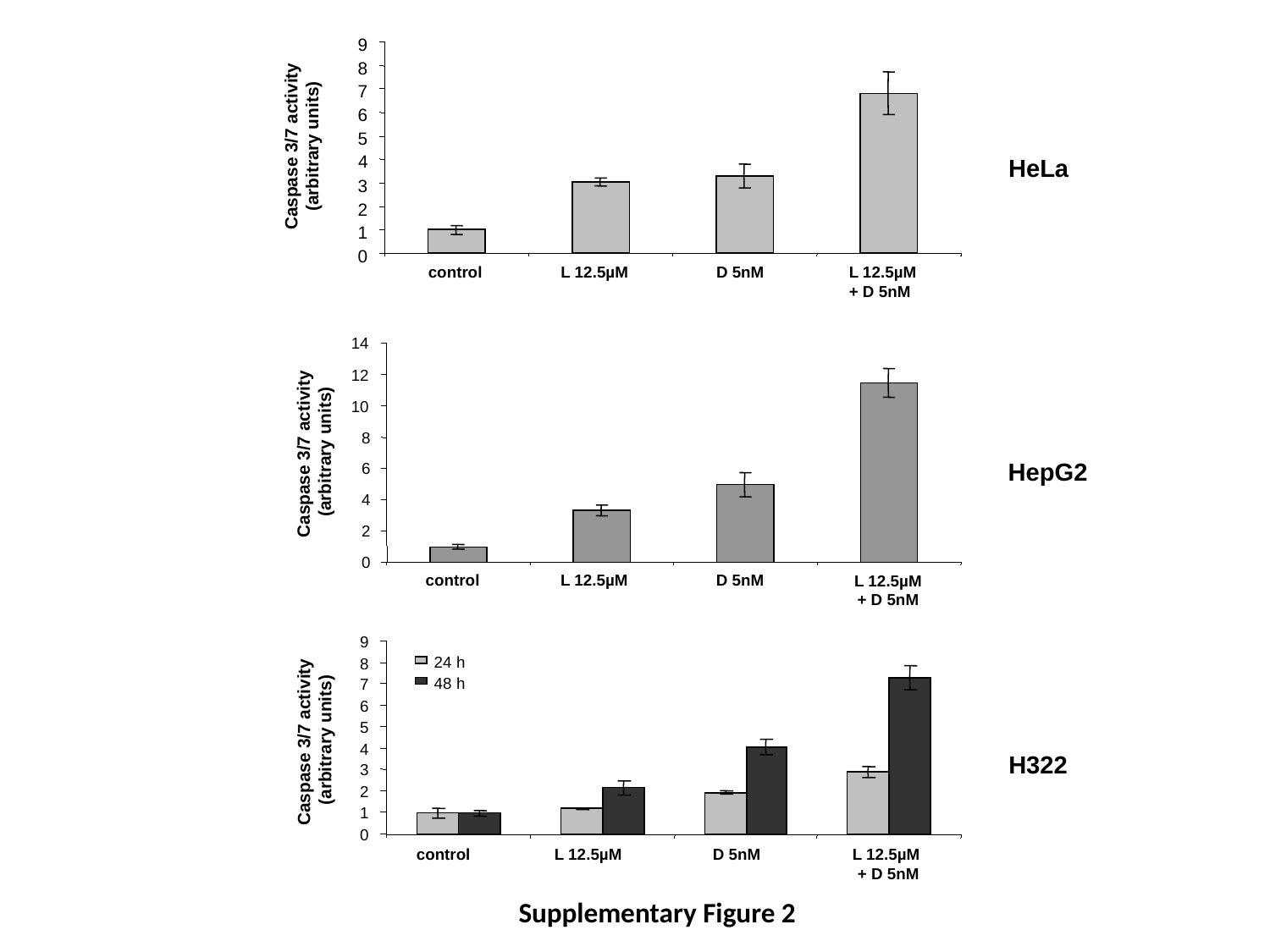

9
8
7
6
5
4
3
2
1
0
 Caspase 3/7 activity
(arbitrary units)
control
L 12.5µM
D 5nM
L 12.5µM
+ D 5nM
HeLa
14
12
10
8
6
4
2
0
Caspase 3/7 activity
(arbitrary units)
control
L 12.5µM
D 5nM
L 12.5µM
+ D 5nM
HepG2
9
8
7
6
5
4
3
2
1
0
24 h
48 h
Caspase 3/7 activity
(arbitrary units)
control
L 12.5µM
D 5nM
L 12.5µM
+ D 5nM
H322
Supplementary Figure 2

## Slide 3
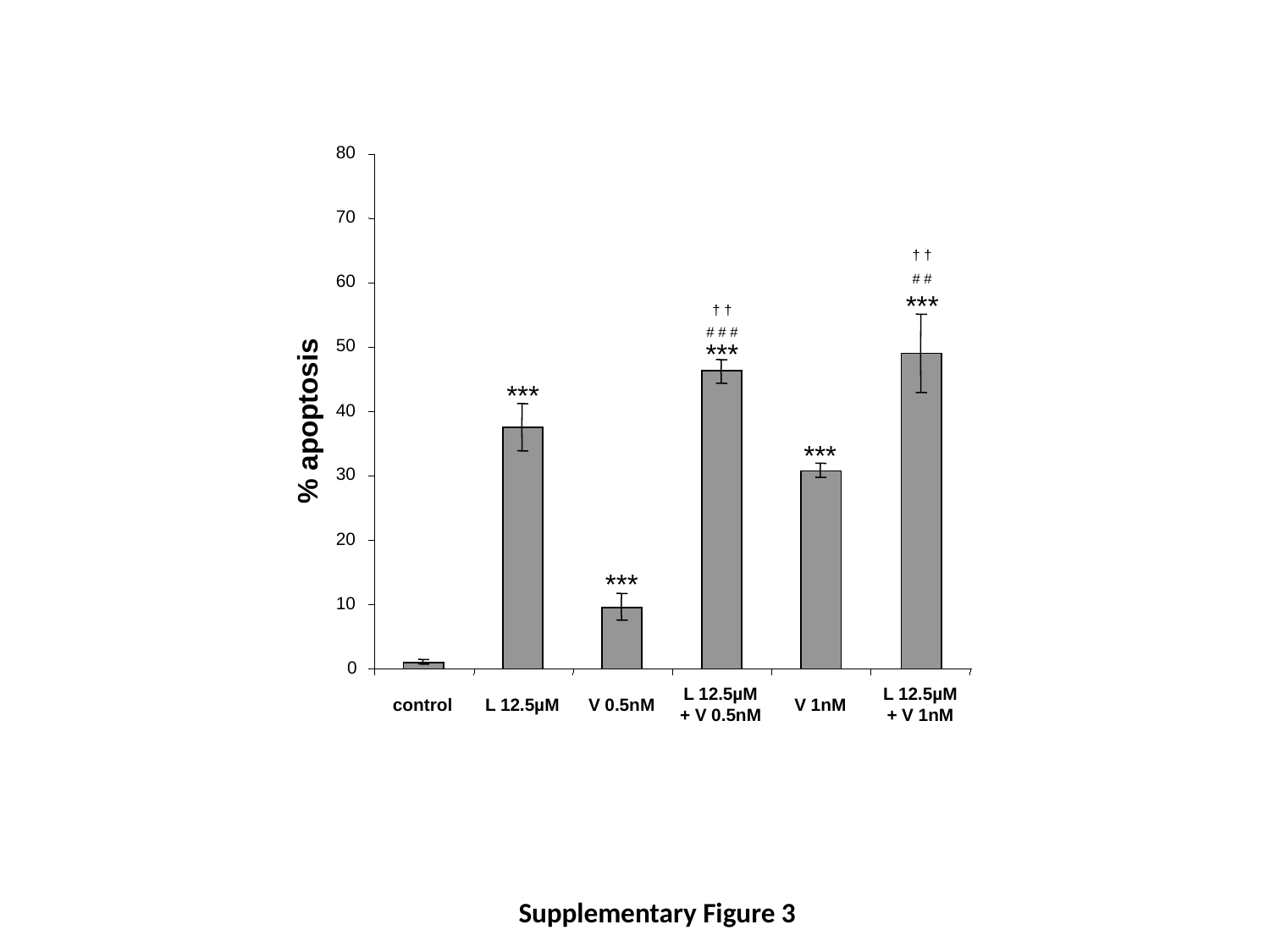

80
70
60
50
40
30
20
10
0
% apoptosis
L 12.5µM
+ V 0.5nM
L 12.5µM
+ V 1nM
control
L 12.5µM
V 0.5nM
V 1nM
† †
# #
***
† †
# # #
***
***
***
***
Supplementary Figure 3

## Slide 4
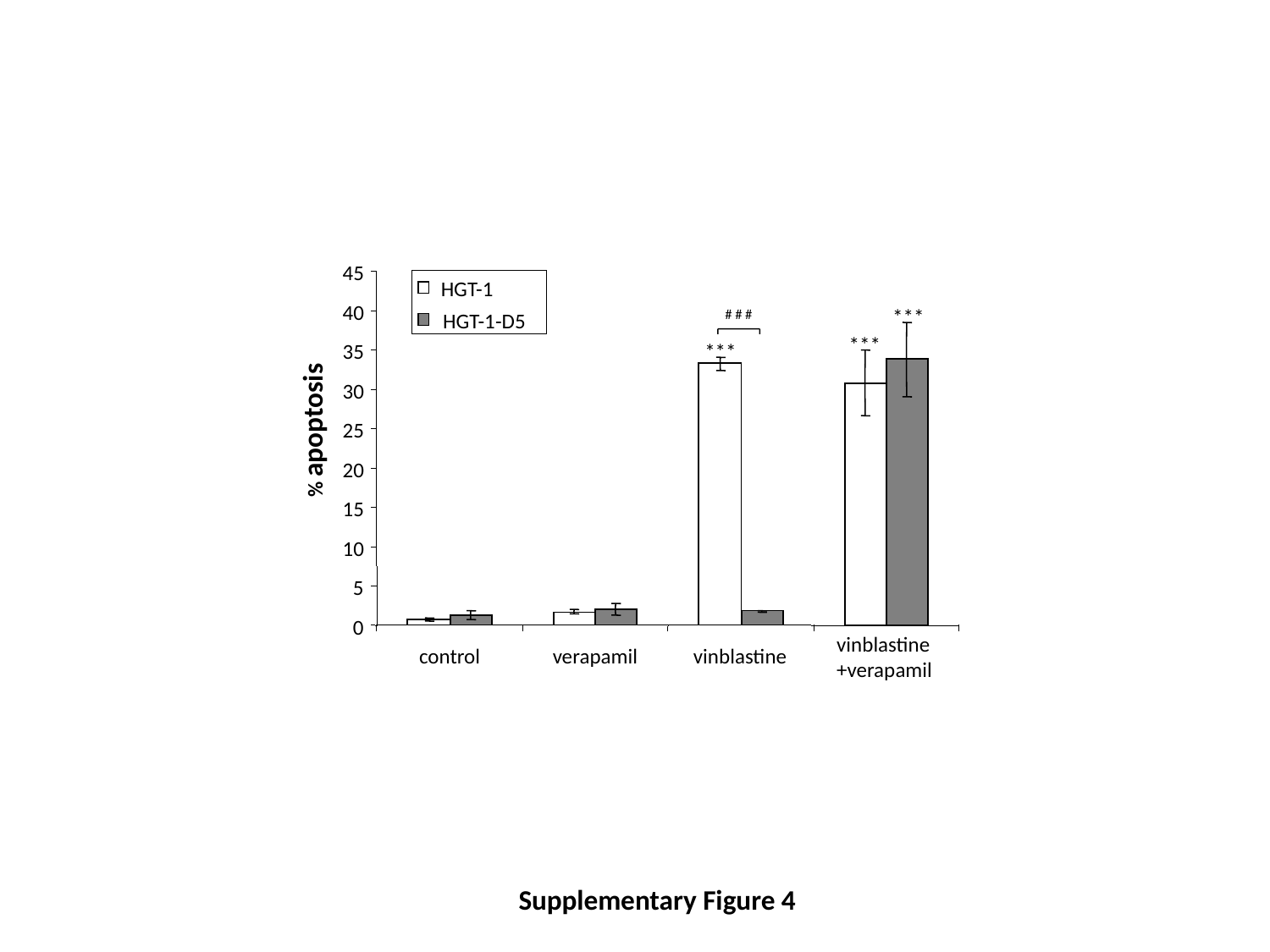

45
HGT-1
HGT-1-D5
40
35
30
% apoptosis
25
20
15
10
5
0
***
# # #
***
***
vinblastine
+verapamil
control
verapamil
vinblastine
Supplementary Figure 4

## Slide 5
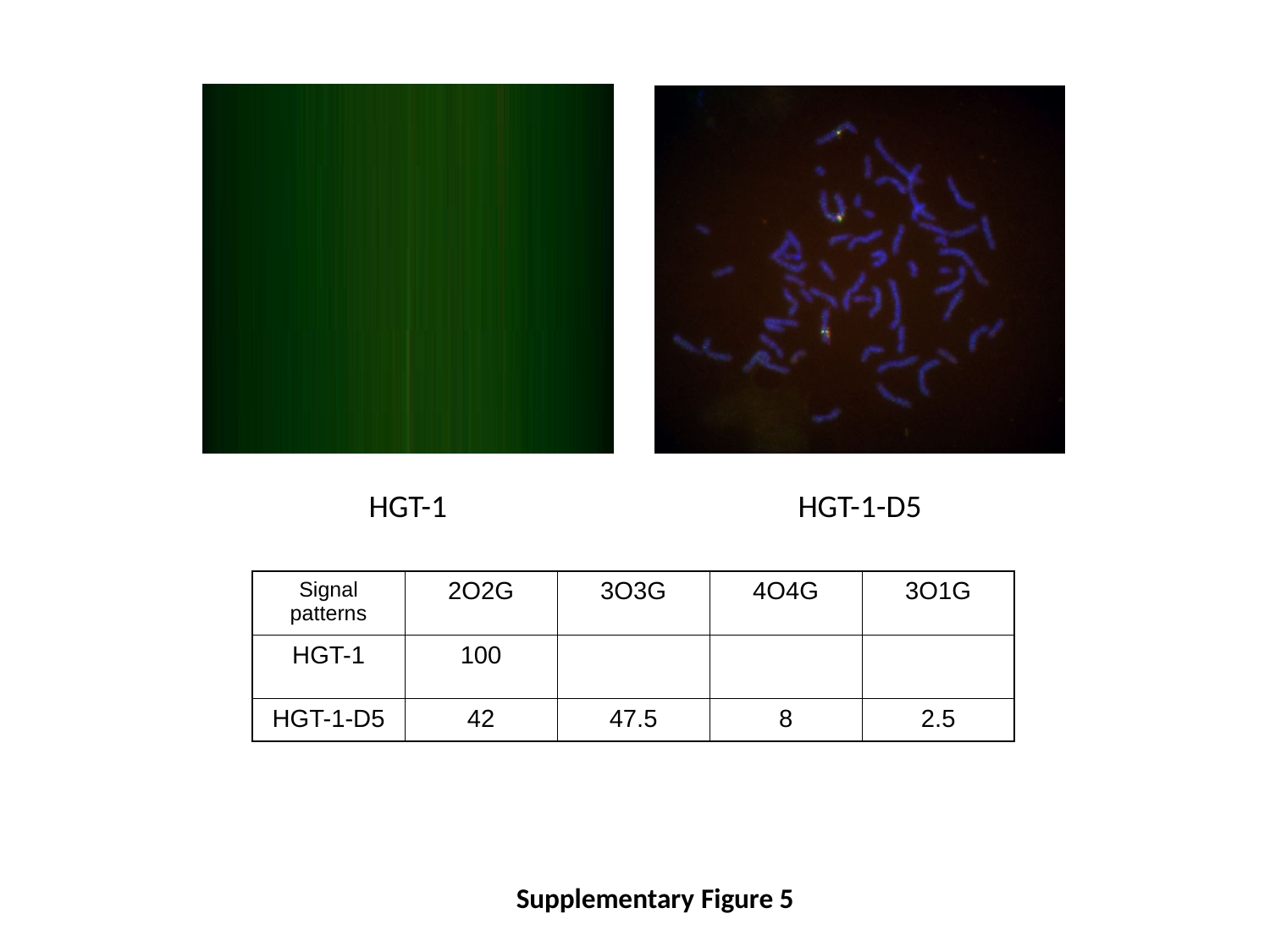

HGT-1
HGT-1-D5
| Signal patterns | 2O2G | 3O3G | 4O4G | 3O1G |
| --- | --- | --- | --- | --- |
| HGT-1 | 100 | | | |
| HGT-1-D5 | 42 | 47.5 | 8 | 2.5 |
Supplementary Figure 5
