# Supplementary material for: The association of statins and taxanes: an efficient combination trigger of cancer cell apoptosis
Source: Br J Cancer. 2012 Jan 31;106(4):685–92. doi: 10.1038/bjc.2012.6 (PMC3322964; doi:10.1038/bjc.2012.6)
Supplement: Supplementary Tables [file bjc20126x2.doc]

SUPPLEMENTARY TABLES

**TABLE 1 : Gene expression changes in response to lovastatin, docetaxel or lovastatin+docetaxel**

| Confidence level  Condition | P<0.05 | P<0.02 | P<0.01 |
| --- | --- | --- | --- |
| Lovastatin |  |  |  |
| Up-regulated genes | 512 | 476 | 362 |
| Down-regulated genes | 768 | 702 | 508 |
| Docetaxel |  |  |  |
| Up-regulated genes | 98 | 41 | 0 |
| Down-regulated genes | 8 | 2 | 0 |
| Lovastatin+Docetaxel |  |  |  |
| Up-regulated genes | 656 | 613 | 499 |
| Down-regulated genes | 764 | 683 | 552 |

**TABLE 2 : Genes involved in the control of lipid metabolism**

| Accession N° | Symbol | Gene | regulation | p-value | Fold change |
| --- | --- | --- | --- | --- | --- |
| NM_005891 | ACAT2 | Acetyl-Coenzyme A acetyltransferase 2 | up | 5x 10^-3^ | 3.7 |
| NM_002130 | HMGCS1 | 3-hydroxy-3-methylglutaryl-Coenzyme A synthase 1 (soluble), transcript variant 2 | up | 5.4x10^-3^ | 4.4 |
| NM_000859 | HMGCR | 3-hydroxy-3-methylglutaryl-Coenzyme A reductase, transcript variant 1 | up | 7.6x10^-3^ | 3.5 |
| NM_000431 | MVK | Mevalonate kinase, transcript variant 1 | up | 2.3x10^-2^ | 1.7 |
| NM_002461 | MVD | Mevalonate (diphospho) decarboxylase | up | 7.9x10^-3^ | 2.5 |
| NM_004508 | IDI1 | Isopentenyl-diphosphate delta isomerase 1 | up | 1.7x10^-2^ | 2.5 |
| NM_002004 | FDPS | Farnesyl diphosphate synthase, transcript variant 1 | up | 2.7x10^-3^ | 2.4 |
| NM_004462 | FDFT1 | Farnesyl-diphosphate farnesyltransferase 1 | up | 1.2x10^-2^ | 2.2 |
| NM_003129 | SQLE | Squalene epoxidase | up | 4.7x10^-3^ | 2.5 |
| NM_002340 | LSS | Lanosterol synthase, transcript variant 1 | up | 8x10^-3^ | 2.2 |
| NM_001001438 | LSS | Lanosterol synthase, transcript variant 2 | up | 1.7x10^-2^ | 2.8 |
| NM_000786 | CYP51A1 | Cytochrome P450, family 51, subfamily A, polypeptide 1, transcript variant 1 | up | 6.9x10^-3^ | 2.8 |
| NM_004837 | GGPS1 | Geranylgeranyl diphosphate synthase 1, transcript variant 1 | up | 1.1x10^-2^ | 1.8 |
| NM_020381 | PDSS2 | Prenyl (decaprenyl) diphosphate synthase, subunit 2 | up | 3.6x10^-2^ | 1.7 |
| NM_198336 | INSIG1 | Insulin induced gene 1, transcript variant 2 | up | 5.4x10^-3^ | 2.7 |
| NM_004599 | SREBF2 | Sterol regulatory element binding transcription factor 2 | up | 3.4x10^-3^ | 1.6 |
| NM_000527 | LDLR | Low density lipoprotein receptor | up | 4.3x10^-3^ | 2.7 |
| NM_004104 | FASN | Fatty acid synthase | up | 1.1x10^-2^ | 3.4 |

TABLE 3 : Genes involved in the control of cell division

| Accession N° | Symbol | Gene | regulation | p-value | Fold change |
| --- | --- | --- | --- | --- | --- |
| NM_033031 | CCNB3 | Cyclin B3, transcript variant 3 | down | 4.6x10^-3^ | 3.3 |
| NM_031966 | CCNB1 | Cyclin B1 | down | 7.6x10^-3^ | 2.4 |
| NM_004701 | CCNB2 | Cyclin B2 | down | 3.5x10^-3^ | 2.3 |
| NM_182851 | CCNB1IP1 | Cyclin B1 interacting protein 1, transcript variant 3 | up | 4.8x10^-3^ | 2.3 |
| NM_001237 | CCNA2 | Cyclin A2 | down | 2.8x10^-2^ | 2.2 |
| NM_002592 | PCNA | Proliferating cell nuclear antigen, transcript variant 1 | down | 4.5x10^-3^ | 2.7 |
| NM_005030 | PLK1 | Polo-like kinase 1 | down | 4.8x10^-3^ | 2.5 |
| NM_001786 | CDK1 | Cyclin-dependent kinase 1, transcript variant 1 | down | 6.3x10^-3^ | 3.2 |
| NM_005192 | CDKN3 | Cyclin-dependent kinase inhibitor 3, transcript variant 1 | down | 5x10^-3^ | 2.3 |
| NM_078626 | CDKN2C | Cyclin-dependent kinase inhibitor 2C (p18, inhibits CDK4), transcript variant 2 | down | 1.1x10^-2^ | 2.1 |
| NM_000389 | CDKN1A | Cyclin-dependent kinase inhibitor 1A (p21, Cip1), transcript variant 1 | up | 7.2x10^-3^ | 2.0 |
| NM_003504 | CDC45L | CDC45 cell division cycle 45-like | down | 8x10^-3^ | 2.8 |
| NM_152562 | CDCA2 | Cell division cycle associated 2 | down | 7.4x10^-3^ | 2.6 |
| NM_031299 | CDCA3 | Cell division cycle associated 3 | down | 1.9x10^-3^ | 2.5 |
| NM_001789 | CDC25A | Cell division cycle 25 homolog A, transcript variant 1 | down | 6.1x10^-3^ | 2.4 |
| NM_080668 | CDCA5 | Cell division cycle associated 5 | down | 4.3x10^-3^ | 2.3 |
| NM_018101 | CDCA8 | Cell division cycle associated 8 (Borealin) | down | 7.1x10^-3^ | 2.3 |
| NM_031299 | CDCA3 | Cell division cycle associated 3 | down | 2.5x10^-3^ | 2.3 |
| NM_001789 | CDC25A | Cell division cycle 25 homolog A, transcript variant 1 | down | 4.3x10^-3^ | 2.2 |
| NM_001790 | CDC25C | Cell division cycle 25 homolog C, transcript variant 1 | down | 9.3x10^-3^ | 2.1 |
| NM_003503 | CDC7 | Cell division cycle 7 homolog, transcript variant 1 | down | 7.8x10^-3^ | 2.1 |
| NM_182751 | MCM10 | Minichromosome maintenance complex component 10, transcript variant 1 | down | 4.8x10^-3^ | 3.1 |
| NM_004526 | MCM2 | Minichromosome maintenance complex component 2 | down | 2.7x10^-3^ | 3.1 |
| NM_006739 | MCM5 | Minichromosome maintenance complex component 5 | down | 4.9x10^-3^ | 2.7 |
| NM_005914 | MCM4 | Minichromosome maintenance complex component 4, transcript variant 1 | down | 4.7x10^-3^ | 2.4 |
| NM_002388 | MCM3 | Minichromosome maintenance complex component 3 | down | 5.9x10^-3^ | 2.1 |
| NM_005915 | MCM6 | Minichromosome maintenance complex component 6 | down | 1.2x10^-2^ | 2.0 |
| NM_001012271 | BIRC5 | Baculoviral IAP repeat-containing 5 (Survivin), transcript variant 3 | down | 3.2x10^-3^ | 3.2 |
| NM_004217 | AURKB | Aurora kinase B | down | 5.2x10^-3^ | 2.6 |
| NM_198433 | AURKA | Aurora kinase A, transcript variant 1 | down | 3.4x10^-3^ | 2.2 |
| NM_004336 | BUB1 | Budding uninhibited by benzimidazoles 1 homolog | down | 5.6x10^-3^ | 2.4 |
| NM_001211 | BUB1B | Budding uninhibited by benzimidazoles 1 homolog beta | down | 1.2x10^-2^ | 2.2 |
| NM_001040694 | INCENP | Inner centromere protein antigens 135/155kDa, transcript variant 1 | down | 1.4x10^-2^ | 2.4 |
