# Supplementary material for: The association of statins and taxanes: an efficient combination trigger of cancer cell apoptosis
Source: Br J Cancer. 2012 Jan 31;106(4):685–92. doi: 10.1038/bjc.2012.6 (PMC3322964; doi:10.1038/bjc.2012.6)
Supplement: Supplementary Figure and Table Legends [file bjc20126x3.doc]

**Legend to supplementary Table 1**

Summary data for the number of genes showing differential expression (with fold change >2) in response to lovastatin (12.5 µM), docetaxel (5 nM) or lovastatin (12.5 µM) + docetaxel (5 nM), after 48h.

**Legend to supplementary Tables 2 and 3**

The tables show the genes which expression was increased (up) or decreased (down) following treatment with lovastatin (12.5 µM), and the associated p-value, after 48h.

**Legends to supplementary Figures**

Supplementary Figure 1

**Time-course and dose-response of apoptosis induction by lovastatin and docetaxel in HGT-1 cells.**

Apoptosis was determined by Hoechst 33342 staining. A) Dose-response and time-course analysis of lovastatin-induced apoptosis. Values are means ±S.D.(n=3). B) Dose-response and time-course analysis of docetaxel-induced apoptosis. Values are means ±S.D.(n=6).

Supplementary Figure 2

**Caspase 3/7 activity induction in other cancer cell lines**

Caspase 3/7 activity (as measured using the Caspase Glo^TM^ 3/7 assay) was determined after 48h in HepG2, after 24h in HeLa cells and after 24h and 48h in H322 cells for 12.5 µM lovastatin (L12.5), 5 nM docetaxel (D5) alone or in combination. The results shown are from one experiment representative of two independent experiments with similar results. Values are means ±S.D. of three wells.

Supplementary Figure 3

**Apoptosis induction by lovastatin and vinblastine in HGT-1 cells**

HGT-1 cells were treated with 12.5 µM lovastatin (L12.5) or with 0.5 or 1 nM vinblastine (V0.5 or V1) alone or in combination for 48h. Apoptosis was determined by Hoechst 33342 staining. Values are means ±S.D. (n=4). * Compared with control, # compared with docetaxel treatment, † compared with lovastatin treatment. Two symbols: *P*<0.01, three symbols: *P*<0.001 (Student’s *t* test).

Supplementary Figure 4

**Effect of vinblastine in HGT-1 and HGT-1-D5 cells**

HGT-1 and docetaxel-resistant HGT-1 cells (named HGT-1-D5) were treated with 1 nM vinblastine alone or in combination with 20 µM verapamil for 48h. Apoptosis was determined by Hoechst 33342 staining. Values are means ±S.D. (n=3). *** *P*<0.001 compared with the control, # # # *P*<0.001 for HGT-1-D5 cells compared with HGT-1 cells (Student’s *t* test).

Supplementary Figure 5

**FISH analysis of MDR-1 gene copies**

Cell metaphases were hybridized with labeled BAC clones. For the HGT-1 cell line, all metaphase cells showed a normal signal pattern for RP11-806M4 and RP11-42N21 probes (two green and two orange signals). For the HGT-1-D5 cell line, 47.50 % of metaphase cells showed three orange and three green signals.
